# 799 A Single Center Retrospective Cohort Study of Burn Scar Alopecia Surgery

**DOI:** 10.1093/jbcr/irae036.339

**Published:** 2024-04-17

**Authors:** Jose Antonio Arellano, Tiffany Jeong, Sumaarg Pandya, Hilary Liu, Mario Alessandri-Bonetti, Guy M Stofman, Francesco Egro

**Affiliations:** University of Pittsburgh Medical Center, Pittsburgh, PA; University of Pittsburgh Medical Center, Pittsburgh, PA; University of Pittsburgh Medical Center, Pittsburgh, PA; University of Pittsburgh Medical Center, Pittsburgh, PA; University of Pittsburgh Medical Center, Pittsburgh, PA; University of Pittsburgh Medical Center, Pittsburgh, PA; University of Pittsburgh Medical Center, Pittsburgh, PA

## Abstract

**Introduction:**

Following acute management and wound healing after burn injuries, burn care may shift toward the aesthetic goals of the patient. Hair restoration is often a multistage process made more complicated by the loss of hair follicles in burn scars, destruction of hair bearing areas, the prioritization of other functional/aesthetic goals, or medical costs. While other studies have compared surgical techniques or management algorithms in burn scar alopecia (BSA) , little research has focused on patient decision to pursue burn scar alopecia treatment. This study aims to describe clinical features of patients who develop BSA and pursue treatment at a single center over 14 years.

**Methods:**

We conducted a retrospective study to review patients with scalp burns from April 2009 and February 2023. Medical records were obtained from patients’ charts.

**Results:**

27 patients had scalp burns. 51.9%(n=14) of patients with scalp burns were female. Most patients suffered thermal injuries (77.8%, n=21). On average, burns encompassed 23.3% ±21.5% TBSA. Most of the burns were full thickness (66.7%, n=18). Patients were followed for 1.9±2.3 years after surgery. Collectively, these patients underwent 69 scalp surgeries. These procedures included excision (39.1%, n=27), debridement (30.4%, n=21), split-thickness skin graft (30.4%, n=21), and tissue expansion (18.8%, n=13). Most of the surgeries were to address BSA (65.2%, n=45).

51.9%(n=14) of patients with scalp burns developed BSA. Most patients who developed BSA (71.4%, n=10) elected to pursue surgical treatment for BSA and received 4.8±3.0 scalp surgeries. Surgical interventions occurred 1.5±3.6 years after burn injury. 33.3% of males and 12.5% of females stopped pursuing treatment for BSA, this difference was not statistically significant(p=0.75). All patients who suffered chemical burns from hair salon accidents developed burn scar alopecia had < 0.5% TBSA burns and pursued surgical treatment(n=4). However, 50%(n=5) of the patients who sought treatment for BSA had severe full-thickness burns >20% TBSA. There was no difference in average TBSA between patients who were treated for BSA and those who were not (p=0.34). However, the average BSA defect size was greater in patients who sought BSA surgery than those who did not (111.4±100.8 cm2 and 24.5±14.6 cm2, p=0.018).

**Conclusions:**

Post-burn trauma aesthetic goals are individualized. While patients often wait years after their scalp burn, most patients pursue multiple surgeries to address BSA. Based on these findings, surgeons should consider burn injury mechanism, timing after burn injury, the financial burden of staged reconstruction, and defect size to facilitate shared decision-making.

**Applicability of Research to Practice:**

BSA may contribute to a negative self-image. These data suggest that patients elect to surgically manage BSA regardless of TBSA and gender, suggesting treatment options for BSA should be widely offered.

